# Metabolic Outcomes After Pancreas Transplant Alone From Donation After Circulatory Death Donors-The UK Transplant Registry Analysis

**DOI:** 10.3389/ti.2023.11205

**Published:** 2023-05-17

**Authors:** Jeevan Prakash Gopal, Adam McLean, Anand Muthusamy

**Affiliations:** ^1^ Imperial College Renal and Transplant Centre, Imperial College Healthcare NHS Trust, Hammersmith Hospital, London, United Kingdom; ^2^ Department of Surgery and Cancer, Imperial College London, London, United Kingdom

**Keywords:** pancreas transplantation, donation after circulatory death, metabolic outcomes, DCD donors, pancreas transplant alone

## Abstract

Extrapolating data from early DCD (donation after circulatory death) kidney transplantation, pancreas transplants from DCD grafts were feared to have worse metabolic outcomes. Hence, we aimed to address the question of pancreas transplant alone (PTA) from DCD donors–are our concerns justified? A UK transplant registry analysis of 185 PTA performed between 2005 and 2018 was done. All early graft losses (<3 months) were excluded to allow focus on the metabolic outcomes (HbA_1_c, weight gain and incidence of secondary diabetic macrovascular complications). The aim was to compare the metabolic outcomes, rejection rates (including the need for steroids), patient and graft survival between DBD (Donation after brainstem death) and DCD groups. After excluding early graft losses, data from 162 PTA (DBD = 114 and DCD = 48) were analyzed. Body mass index of the donor was less in DCD group (DBD = 23.40 vs. DCD = 22.25, *p* = 0.006) and the rest of the baseline transplant characteristics were comparable. There were no significant differences in the HbA_1_c, weight gain, rejection rate, and incidence of secondary diabetic macrovascular complications post-transplant between DBD and DCD recipients. The 1-, 5-, and 10-year patient and graft survival were similar in both the groups. PTA from DCD donors have equivalent metabolic outcomes and survival (patient/graft) as that of DBD donors.

## Graphical Abstract

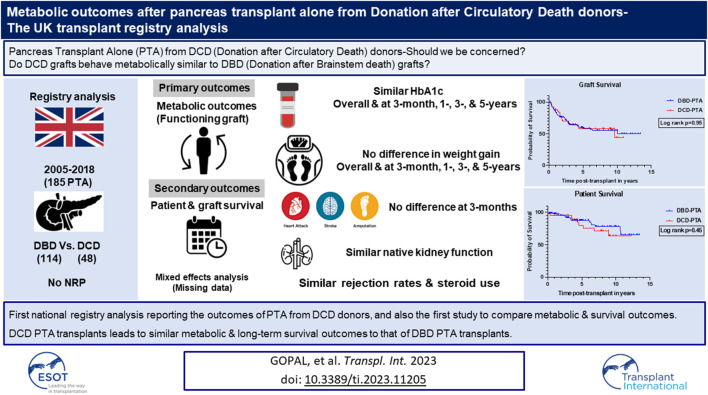

## Introduction

Despite the increasing awareness regarding organ donation, scarcity of suitable donor organs is still a problem faced by the transplant community. The median months to transplantation for a pancreas transplant alone (PTA) in the US was 24.1 months in 2016–2017 [1]. In the Euro transplant region, 75% of the patients were still waiting for a pancreas transplant at 1 year from listing [2]. The waiting list mortality is still significant. In the US, wait list mortality for PTA was 2.7% per 100 patient-years in 2019 and in the UK and Euro transplant region, the waitlist mortality was 3% within 1 year of listing [1–3].

Transplanting DCD organs has been a viable option to expand the donor pool. The UK has pioneered pancreas transplantation from DCD donors and in recent years about one-third of pancreas transplants performed are from controlled DCD donors, and the pancreas offer decline rate is better for DCD organs (45%) than DBD organs (54%) [3]. While the UK has been a global leader in DCD pancreas transplantation, DCD pancreas utilization rate in the US and Euro transplant region has remained low. In the US, less than 5% of the pancreas transplanted were from DCD donors and it has remained consistently low since 2008 [1]. A similar picture is noted in the Euro transplant region [2]. The main concerns for the differential usage are functional warm ischemia time and asystolic period prior to commencement of organ perfusion with the resultant ischemia reperfusion injury and consequent graft pancreatitis, sepsis and graft thrombosis.

Convincing evidence supporting DCD pancreas transplantation is being generated since 2000 [4–11]. None of the studies have looked into the metabolic outcomes and as such the metabolic outcomes after PTA from DCD grafts are unknown. A successful pancreas transplant, unlike intensive insulin regimen, restores euglycemia without the risk of hypoglycaemia and halts or reverses the progression of secondary complications of diabetes [12–14]. Hence, the real premise of pancreas transplantation especially in the setting of PTA is to achieve optimal metabolic control in addition to achieving insulin independence.

In patients with diabetes, chronic hyperglycaemia is known to be associated with an increased risk of cardiovascular disease, whereas, in patients without diabetes a higher HbA1c even within the normal range is associated with a significantly higher risk of coronary artery disease [15, 16]. This highlights the importance of stricter glycaemic control to achieve the maximum benefit. In addition, early post-transplant impaired glucose tolerance is associated with later pancreas graft failure [17]. Hence, it is vital to know the metabolic outcomes. Therefore, we aimed to study the metabolic outcomes after PTA and compare between DBD and DCD grafts.

## Materials and Methods

### Data Collection

There are eight designated pancreas transplant centres in the UK and all of them report their follow up data to the UK Transplant Registry, which is a mandatory prospectively run database maintained by the National Health Service Blood and Transplant (NHSBT). The Pancreas Advisory Group, a subsection of NHSBT approved this study and provided access to the data. All patients who underwent PTA in the UK from 1 January 2005 to 31 December 2018 were identified, and pertinent data was obtained from the UK Transplant Registry. HbA_1_C was recorded as % prior to 2013 and as mmol/mol since then.

### Indications and Restrictions for PTA

All patients waitlisted for PTA had insulin treated diabetes along with normal or near-normal renal function. They also had at least 2 severe hypoglycaemic episodes within the last 24 months and assessed by a diabetologist to have disabling hypoglycaemia. Body mass index (BMI) > 30 kg/sq.m for patients with type 2 diabetes was an absolute contraindication for PTA, whereas, insulin requirement >100 units/day, BMI > 30 kg/sq.m, and severe aorto-iliac or peripheral vascular disease were relative contraindications. The rest of the contraindications were similar to most of the solid organ transplants and are described elsewhere [18].

### Donor Selection

The donor selection criteria were uniform across all pancreas transplant centres. The criteria were similar for DBD and DCD donors except for age (DCD ≤ 55 years of age; DBD ≤ 60 years of age). The following were contraindications to pancreas donation: history of diabetes in the donor (excludes insulin requirement in critical care), active or previous pancreatitis, previous pancreatic surgery, body mass index (BMI) > 40 kg/m^2^, weight < 15 kg, as well as other contraindications for solid organ transplantation. The UK pancreas offering scheme has been national since 2010, with patient-specific offers and a combined list for solid organ pancreas and islet transplants. The national pancreas allocation scheme is described elsewhere [19]. All the waiting list patients were considered suitable for DCD organs without any distinction.

### DCD Pancreas Procurement

In April 2010, the National Organ Retrieval Service (NORS) was established to carry out all the organ retrievals in the UK and before that, the corresponding implanting centres procured their own organs. At present, there are 10 abdominal NORS teams in the UK (six teams are associated with a pancreas transplant program) and eight teams are on-call on any given day. Depending on the location of the donor hospital and the availability of the nearest NORS teams, an appropriate NORS team will be mobilized for retrieval. Pre-mortem interventions such as heparinization or vascular cannulation are prohibited in the UK and organs were retrieved only from controlled DCD donors using a super-rapid technique. After obtaining informed consent from the next of kin, withdrawal of life-sustaining treatment (WLST) occurred either in the critical care unit or in the anaesthetic room of the operating theatre depending on the local hospital policy. After WLST, NORS team wait for 3 h for the onset of functional warm ischemia (defined as systolic blood pressure < 50 mmHg). Pancreases were procured if donor asystole occurred within 30 min following the onset of functional warm ischemia. NORS team will abandon pancreas procurement if asystole does not occur within the above time frame. There is a mandatory 5-min period following donor asystole before death can be declared and subsequently another 5-min “No touch” period following declaration of death and prior to commencement of organ procurement. Through a midline laparotomy, the donor distal aorta or common iliac vessels were cannulated and *in-situ* perfusion was commenced with University of Wisconsin solution (ViaspanTM, Bristol-Myers Squibb Pharmaceuticals, Uxbridge, United Kingdom). If the liver was procured, portal venous cannulation was performed with proximal venting of the portal vein. Normothermic regional perfusion (NRP) was not utilized in this study population.

### Retrieval Training

Retrieval competency is governed by NHSBT. In order to gain competency in pancreas retrieval, trainee surgeons enter supervised training in any one of the commissioned NORS teams in the UK and will need to demonstrate appropriate knowledge, skills and attitudes which are compatible with unsupervised retrieval practice. The local NORS lead will be responsible to decide when the trainee surgeon is ready for unsupervised practice. Prior to unsupervised practice, all retrieval-related training and masterclass must be completed. A complete guidance for retrieval training is described elsewhere [20].

### Pancreas Transplantation

Implantation techniques were as per the discretion of the implanting centre/recipient surgeon and both DBD and DCD organs were treated similarly. Immunosuppression protocol were according to the local practice in different centres.

### Outcomes Studied

The primary aim was to compare the metabolic outcomes (HbA1c, weight gain, and secondary diabetic macrovascular complications) between the two groups and it was studied only in recipients with a functioning graft. The metabolic outcomes were compared alongside with the incidence of rejection episode and steroid usage. All the early graft losses (<3 months) were excluded when analysing the metabolic outcomes. The cut off for early graft loss was set at 3 months based on literature evidence [21–23]. When analysing metabolic outcomes failed grafts were excluded (censored at the point of graft failure). The secondary aim was to compare the survival outcomes (both graft and patient) between the two groups.

### Definitions

Functioning graft was defined as being insulin independent post-transplantation. Secondary diabetic macrovascular complications were defined as any of the following events post-transplant: cerebrovascular accident, myocardial infarction, or limb amputation (minor or major). Recipients were classified based on the calculated reaction frequency (cRF) as either sensitised (cRF > 5%) or highly sensitised (cRF > 85%).

### Statistical Analyses

Categorical variables are expressed as frequency (%) and continuous variables as median and interquartile range (IQR). Difference between the categorical variables were assessed by using Fisher’s exact test and chi-squared test. Difference between the continuous variables were assessed by using Mann-Whitney test. The mixed-effects model approach was used in order to obtain unbiased results due to missing observation. The mixed-effects model for repeated observations was constructed without assuming sphericity of the data and performed without any interaction analysis or multiple comparisons. In the mixed-effects model, recipients with a functioning graft at 3 months had longitudinal HbA1c and weight gain data inputted whereas longitudinal serum creatinine data was inputted irrespective of the graft function. Kaplan-Meir survival plots were used for survival analysis. For graft survival, censoring was done for grafts functioning at the time of analysis and death with a functioning graft. All the statistical analyses were performed using Graph Pad Prism software (Version 9.5.1).

## Results

In the study period 185 PTA’s were performed. All early graft losses were excluded at all follow up time points (*n* = 23; DBD = 16/DCD = 7) to allow focus on metabolic outcomes. The early graft losses were included in survival analysis and there were no patient deaths in this group. Out of the 162 PTA’s that were included, 114 were from DBD donors and 48 from DCD donors. The median follow-up period was 4.4 years (IQR: 2.1–8 years). The median asystolic period (downtime) for DCD donors was 11 min (Range: 5–30; *n* = 23). The median withdrawal time (time from withdrawal of life support to circulatory arrest) for DCD donors was 14 min (Range: 0–19; *n* = 11). The median functional warm ischemia time for DCD donors was 16 min (Range: 8–29; *n* = 29).

### Baseline Characteristics

Donor, recipient and transplant characteristics as described in Tables 1–3. Apart from a lower BMI, the rest of the DCD donor characteristics (age, abdominal girth, sex, and ethnicity) were equivalent to DBD donors. Recipient characteristics (age, BMI, sex, ethnicity, duration of diabetes, HbA_1_C at the time of registration, insulin use at the time of registration, and sensitization) and transplant characteristics (level of HLA mismatch, cold ischemia time, anastomosis time, exocrine drainage technique, induction immunosuppression, and proportion of re-transplants) were comparable between the two groups. 93% of the recipients were patients with type 1 diabetes mellitus (*n* = 150). Among the remaining 12 recipients, 7 had type 2 diabetes mellitus (DCD = 1; DBD = 6) and 5 had mixed (type 1 and 2) diabetes mellitus (DCD = 3; DBD = 2). A consistent proportion of PTA’s were performed using DCD grafts across all eras.

**TABLE 1:** Comparison of donor characteristics.

Donor characteristics	Variable	DBD (N = 114)	DCD (N = 48)	*p*-value
Donor age, years		33 (21–48)	29 (18.5–43.8)	0.11
	Missing	0	0	
Donor BMI, kg/sq.m		23.4 (21.1–24.9)	22.2 (19.5–23.8)	0.006
	Missing	0	0	
Donor abdomen girth, cms		84 (76–92)	81.5 (74.2–85)	0.14
	Missing	23	16	
Donor sex	Male	56 (49%)	27 (56%)	0.49
	Female	58 (51%)	21 (44%)	
	Missing	0	0	
Donor ethnicity	Caucasian	104 (91%)	44 (92%)	0.99
	Non-Caucasian	10 (9%)	4 (8%)	
	Missing	0	0	

**TABLE 2:** Comparison of recipient characteristics.

Recipient characteristics	Variable	DBD (N = 114)	DCD (N = 48)	*p*-value
Recipient age, years		41 (34.8–48)	43 (35.3–49.8)	0.63
	Missing	0	0	
Recipient BMI, kg/sq.m		24.7 (22.5–26.9)	24.4 (22.3–30.8)	0.62
	Missing	34	9	
Recipient sex	Male	41 (36%)	18 (38%)	0.85
	Female	73 (64%)	30 (62%)	
	Missing	0	0	
Recipient ethnicity	Caucasian	109 (96%)	47 (98%)	0.67
	Non-Caucasian	5 (4%)	1 (2%)	
	Missing	0	0	
Duration of diabetes (pre-transplant), years		26 (25.2–29.3)	28 (25.5–31.7)	0.54
	Missing	7	2	
Recipient HbA_1_C at registration, mmol/mol		76 (62.9–92.1)	75 (61.6–91.8)	0.52
	Missing	20	2	
Recipient insulin use at registration, IU/day		40 (30–55)	40 (31.3–49.3)	0.80
	Missing	21	8	
Calculated Reaction Frequency, CRF	<85%	104 (91%)	43 (90%)	0.77
	>85%	10 (9%)	5 (10%)	
	Missing	0	0	

**TABLE 3:** Comparison of transplant characteristics.

Transplant characteristics	Variable	DBD (N = 114)	DCD (N = 48)	*p*-value
Era of transplantation	2005–2009	36 (32%)	18 (37%)	0.76
	2010–2014	53 (46%)	20 (42%)	
	2015–2018	25 (22%)	10 (21%)	
Level of HLA mismatch	Level 1	4 (4%)	2 (4%)	0.25
	Level 2	13 (11%)	4 (8%)	
	Level 3	42 (37%)	11 (23%)	
	Level 4	55 (48%)	31 (65%)	
Cold ischemia time, mins		688 (548.5–781.5)	720 (578.0–832.0)	0.19
	Missing	14	9	
Anastomosis time, mins		33 (27–40.2)	37.5 (31.5–44)	0.05
	Missing	20	6	
Exocrine drainage technique	Enteric	64 (56%)	30 (63%)	0.19
	Bladder	38 (33%)	17 (35%)	
	Missing	12 (11%)	1 (2%)	
Induction immunosuppression	Depleting agent	93 (82%)	41 (85%)	0.60
	Non-depleting agent	20 (17%)	6 (13%)	
	Missing	1 (1%)	1 (2%)	
Re-transplants		10 (9%)	2 (4%)	0.30

### Metabolic Outcomes

**FIGURE 1:**
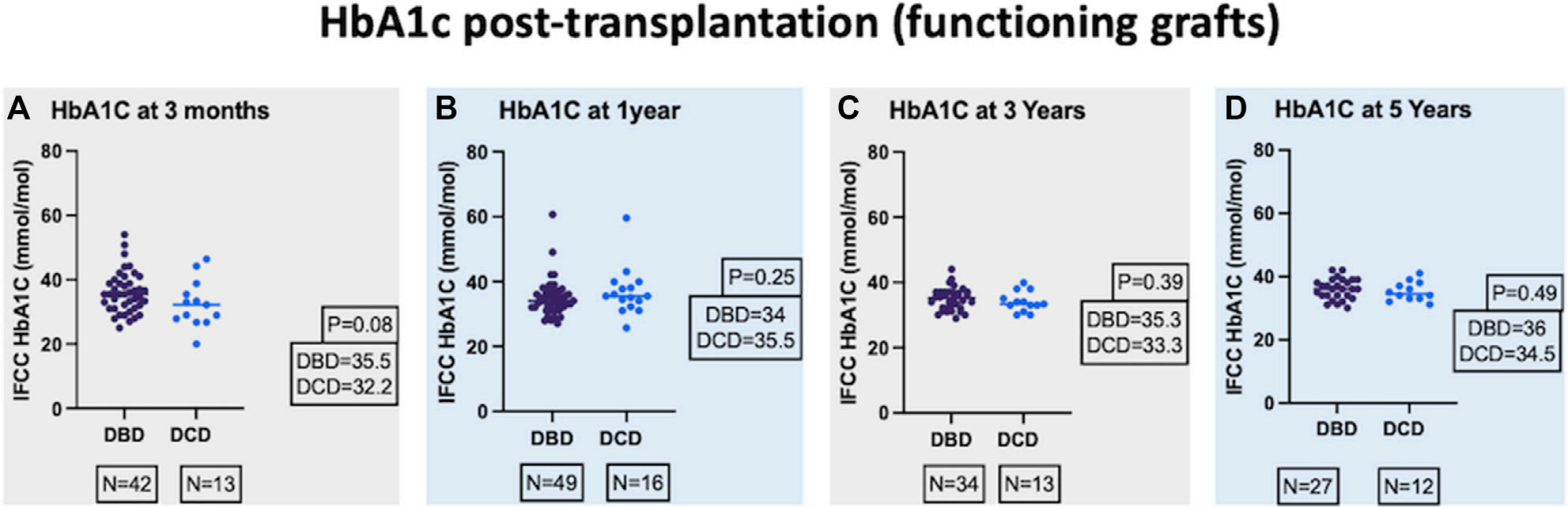
Comparison of IFCC (International Federation of Clinical Chemistry) HbA1c at 3-month **(A)**, 1-year **(B)**, 3-year **(C)**, and 5-year **(D)** post-transplant.

DBD and DCD recipients had similar median post-transplant HbA_1_c millimole/mole at 3-month [35.5 (31.1–39.1) and 32.2 (27.3–37.1)], 1-year [34 (32–37) and 35.5 (32.6–39.4)], 3-year [35.3 (32–37) and 33.3 (32–36.5)], and 5-year post-transplant [36 (34–39) and 34.5 (33–37.7)] and the respective *p* values were 0.08, 0.25, 0.39, and 0.49 (Figure 1). The median HbA1c values in % for DBD and DCD recipients were also equivalent at 3-month [5.4 (5–5.7) and 5.1 (4.6–5.5)], 1-year [5.3 (5.1–5.5) and 5.4 (5.3–5.8)], 3-year [5.4 (5.1–5.5) and 5.2 (5.1–5.6)], and 5-year post-transplant [5.4 (5.2–5.7) and 5.3 (5.1–5.6)] and the respective *p* values were 0.09, 0.25, 0.69, and 0.50. In a mixed-effects model, there was no significant difference in the overall predicted mean HbA1c (millimole/mole) until 5 years post-transplant between the 2 groups (DBD = 39 and DCD = 37, *p* = 0.19).

HbA1c was also compared between the waitlisted candidates for PTA at the time of registration (*n* = 145) and those with a failed PTA graft with HbA1c recorded at the time of graft failure (*n* = 14). There was no significant difference in the median HbA1c (millimole/mole) between the groups [Waitlisted = 76 (63–91) Vs. Failed graft = 60.1 (48–114.3); *p* = 0.35].

**FIGURE 2:**
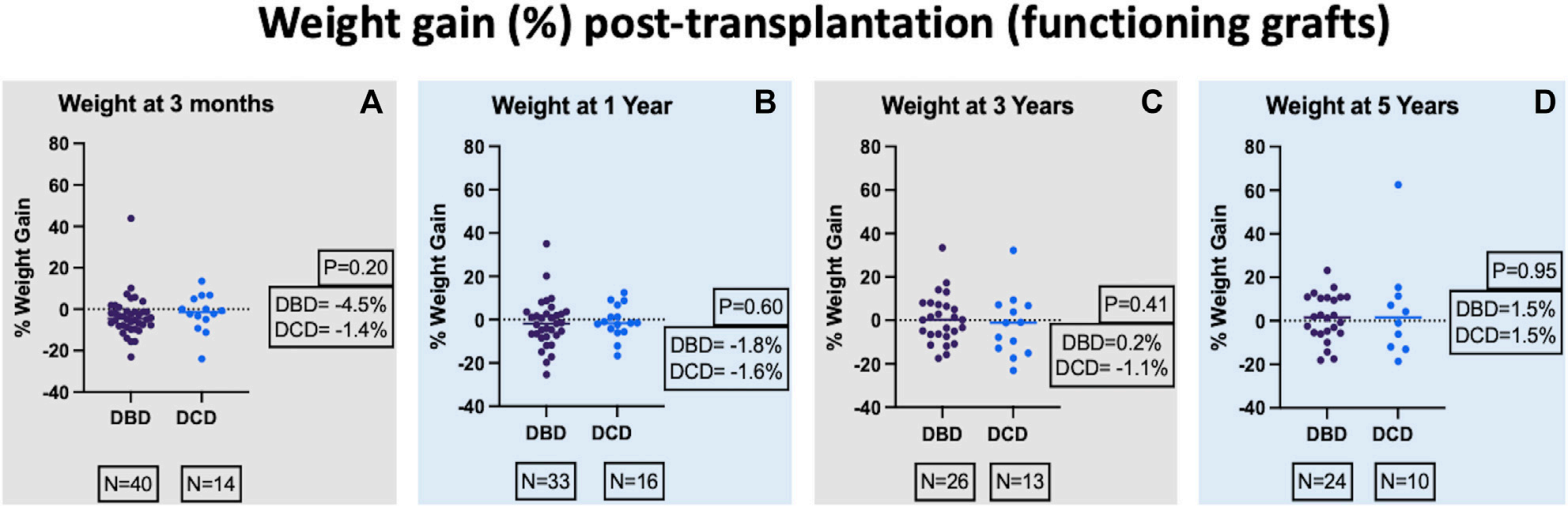
Comparison of percentage weight gain at 3-month **(A)**, 1-year **(B)**, 3-year **(C)**, and 5-year **(D)** post-transplant.

Percentage weight gain post-transplant was calculated (weight post-transplant minus weight pre-transplant/100) and compared between the two groups. There was no significant difference in weight (median percentage weight gain) between the two groups at 3-month, 1-year, 3-year, and 5-year post-transplant and the respective *p* values were 0.20, 0.60, 0.41, and 0.95 (Figure 2). In a mixed-effects model, there was no significant difference in the overall predicted mean percentage weight gain until 5 years post-transplant between the 2 groups (DBD = 6.5 and DCD = −0.8, *p* = 0.86).

The incidence of secondary diabetic complications post-transplant was not significantly different between both the two groups at 3-month post-transplant (DBD = 1% vs. DCD = 2%, *p* = 0.51). There were no secondary diabetic complications in both the groups at 1-year, 3-year, and 5-year post-transplant.

There was no significant difference in the incidence of rejection between the two groups at 3-month (DBD = 10% vs. DCD = 13%, *p* = 0.63), 1-year (DBD = 19% vs. DCD = 10%, *p* = 0.15), 3-year (DBD = 12% vs. DCD = 10%, *p* = 0.71), and 5-year post-transplant (DBD = 10% vs. DCD = 10%, *p* = 1).

The overall steroid free maintenance rate was similar irrespective of the graft type (DBD = 75% vs. DCD = 73%, *p* = 0.79).

HbA_1_C and weight were compared between DBD and DCD grafts in Era 1 (2005–2009) and Era 2 (2010–2014). In both the eras, there was no significant difference in HbA_1_c or weight gain between the two groups at 3-month, 1-year, 3-year, and 5-year post-transplant (Table 4). In Era 3 (2015–2018) 18 PTA’s were performed and out of which only 3 were performed utilizing DCD graft. Follow up data for analysing 3-year and 5-year outcomes was not available. Hence, it was not possible to compare Era 3 metabolic outcomes separately.

**TABLE 4:** Comparison of metabolic outcomes based on era of transplantation.

Outcome/Era	Time post-transplant	DBD	DCD	*p*-Value
HbA_1_C (mmol/mol)/Era-1	3-month	35.5 (31.1–36.6)	30.6 (27.1–38)	0.25
	1-year	34 (31.1–35.5)	35.5 (31.6–39.9)	0.44
	3-year	33.3 (30.6–36.1)	33.2 (30.8–38.2)	0.84
	5-year	36 (31.6–38)	36 (31.6–38.5)	0.89
Weight gain (%)/Era-1	3-month	−4.8 (−8.0 to −0.1)	−0.3 (−3.7–5.3)	0.05
	1-year	−6.6 (−11.0 to 2.4)	−1.6 (−4.2 to 1.2)	0.12
	3-year	−3.5 (−10.8 to 2.8)	1.3 (−10.9–13.4)	0.56
	5-year	−0.5 (−5.9–10.9)	−1.0 (−13.0 to 7.1)	0.57
HbA_1_C (mmol/mol)/Era-2	3-month	37 (31.5–41.5)	33 (24.5–38.8)	0.32
	1-year	34 (32.2–36.7)	35.8 (33.2–39.2)	0.40
	3-year	36 (34–37)	34 (32–36.5)	0.16
	5-year	37 (34–39)	34 (33–37)	0.12
Weight gain (%)/Era-2	3-month	−6.6 (−8.7 to −2.0)	−6.7 (−20.8 to 4.4)	0.92
	1-year	1.0 (−4.8–5.7)	−0.9 (−14.4 to 9.6)	0.74
	3-year	5.7 (0.5–12.3)	−7.8 (−17.3 to 6.7)	0.07
	5-year	1.9 (−1.1–9.7)	11.3 (−11.9–15.3)	0.54

There was no significant difference in the median serum creatinine (micromole/L) between the DBD and DCD recipients at 3-month [104 (76–140) and 104 (82.7–140)], 1-year [107 (80–133) and 108 (80.2–153.5)], 3-year [115.5 (93.5–147) and 114 (96.2–140.8)], and 5-year [127 (96–162.3) and 110 (96–140.5)] post-transplant and their respective *p* values were 0.56, 0.57, 0.83, and 0.51. In a mixed-effects model, there was no significant difference in the overall predicted mean serum creatinine (micromole/L) until 5 years post-transplant between the two groups (DBD = 129.5 and DCD = 133.2, *p* = 0.74).

**FIGURE 3:**
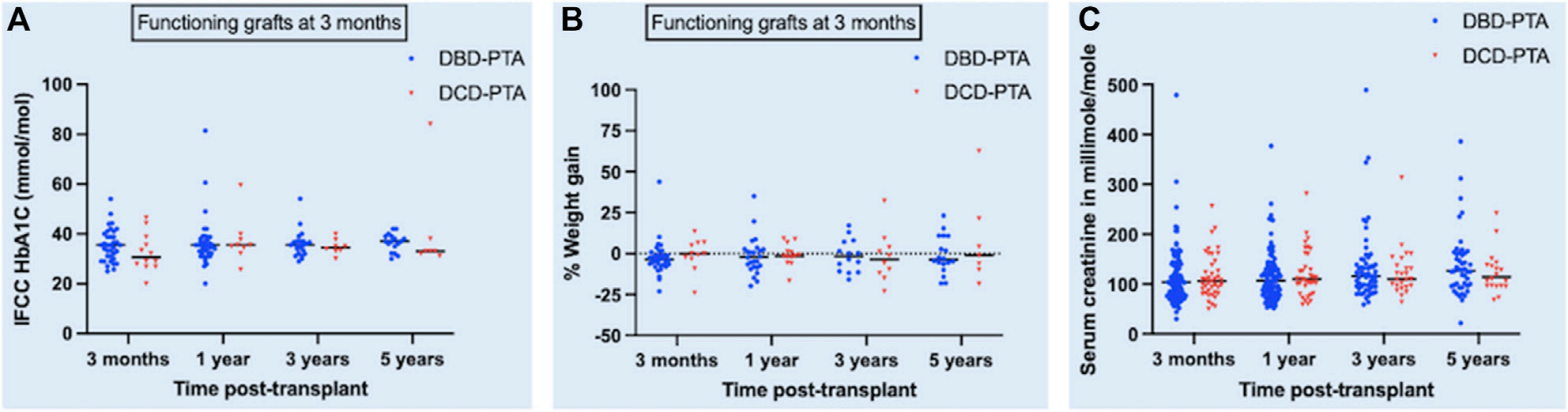
Scatter dot plot showing the evolution of difference in HbA1c [panel **(A)**], percentage weight gain [panel **(B)**] and serum creatinine [panel **(C)**] post-transplant between DBD and DCD groups. Black horizonal line in the plot represents median of each dataset.

The evolution of the difference in HbA1c, weight gain, and serum creatinine between the two groups is shown in the scatter dot plot (Figure 3).

### Survival Outcomes

**FIGURE 4:**
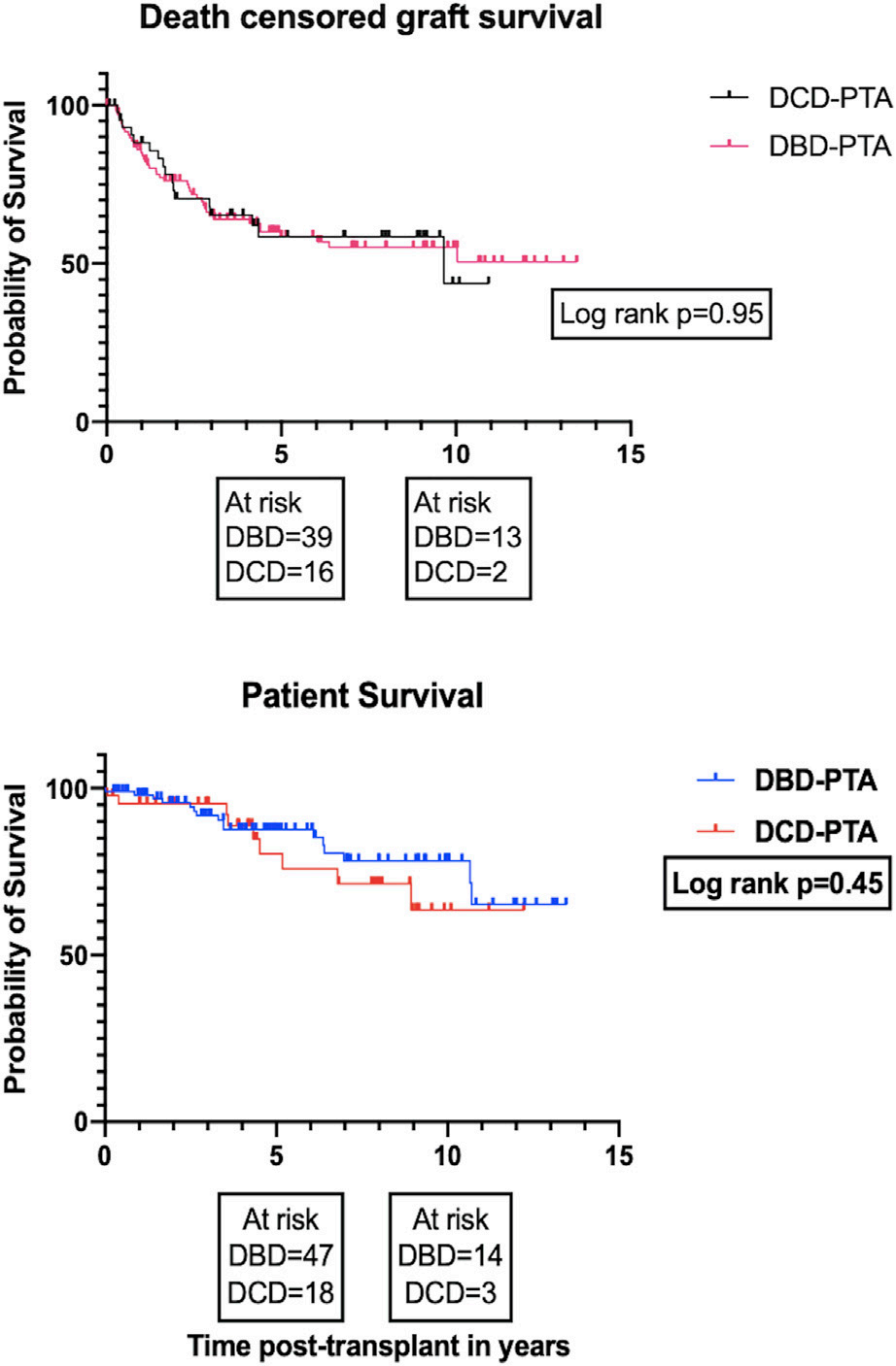
Kaplan Meier plots for death-censored pancreas graft survival and patient survival, respectively.

On univariate analysis, there was no significant difference in the overall death -censored pancreas graft survival and overall patient survival between the DBD and DCD recipients (Figure 4; log rank *p* = 0.95 and *p* = 0.45, respectively). The 1-, 5-, and 10-year patient survival was 98%, 88%, 78% for DBD and 95%, 85%, 63% for DCD recipients. The 1-, 5-, and 10-year death-censored graft survival was 86%, 59%, 53% for DBD and 88%, 59%, 44% for DCD recipients. The proportion of early graft loss was also similar between the two groups. Data pertaining to early graft loss was not part of the study and hence a detailed analysis of the causes for early graft loss was not possible.

### Missing Outcome Data

Graft function was not available for 30 patients (DBD = 20; DCD = 10) at 3-month and 38 patients at 1-year post-transplant (DBD = 23; DCD = 15). Among those with a functioning graft, HbA_1_c data was not available for 73 patients at 3-month (DBD = 49; DCD = 24), 41 patients at 1-year (DBD = 26; DCD = 15), 26 patients at 3-year (DBD = 19; DCD = 7), and 18 patients at 5-year post-transplant (DBD = 13; DCD = 5). Pre-transplant weight was not available for 29 patients (DBD = 24; DCD = 5). Among those with a functioning graft, percentage weight gain data was not available for 74 patients at 3-month (DBD = 51; DCD = 23), 57 patients at 1-year (DBD = 42; DCD = 15), 34 patients at 3-year (DBD = 27; DCD = 7), and 23 patients at 5-year post-transplant (DBD = 16; DCD = 7).

## Discussion

This is the first study comparing metabolic outcomes alongside survival outcomes between DBD and DCD PTA recipients. Post-transplant HbA1c, in addition to being a marker of graft function, is also known to be an independent predictor of pancreas graft failure [24]. Hence it is vital to consider HbA1c alongside survival outcome. We noted comparable HbA1c for functioning DBD and DCD grafts at all time points. The University of Wisconsin have published similar results at 1-year post transplant but their DCD PTA group had only four patients [7]. In terms of weight gain post-transplant among functioning grafts, there was no significant difference between the two groups at all time points. Both the groups lost weight until 1 year and then started to gain weight in spite of similar HbA1c, rejection rates and steroid usage. The weight gain outcome could have been biased by the missing data. Excessive weight gain post pancreas transplantation especially in the intermediate term have been reported in the setting of simultaneous pancreas kidney transplantation [25, 26]. Post-transplant weight gain has reported to be associated with the development of post pancreas transplant diabetes mellitus [27]. There is no literature evidence on weight gain after pancreas transplantation based on graft type. Weight gain has been included in the analysis of metabolic outcomes as treatment of rejection can lead to excessive weight gain and excessive weight gain can influence glycaemic control. Longer term follow-up could uncover the longitudinal trend of weight gain and its consequences such as post-transplant metabolic syndrome and cardiovascular complications.

When comparing the metabolic outcomes, rejection episodes were considered alongside, as treatment of rejection would influence the metabolic parameters. It is not uncommon to treat rejection episodes based on clinical suspicion and hence, it is difficult to distinguish whether these were actual rejection episodes or graft pancreatitis as both of them present with a similar clinical picture. The need for *de novo* steroids post-transplant were considered as a surrogate for rejection but whether steroids were introduced as part of modulation of the immunosuppression regime to counteract infections remain unknown. The incidence of rejection was similar in both the groups at all time points and this was in a setting where the HLA mismatch and the use of depleting agent for induction were similar as well. 73% of the DCD recipients were on steroid-free maintenance immunosuppression and was similar to DBD recipients. The rejection episodes reported to the UK Transplant registry were not classified as cellular or antibody mediated or mixed rejection and hence, an in-depth analysis was not possible.

It is well known that a successful pancreas transplant halts or reverses the progression of secondary diabetic complications [28, 29]. In addition, clinical trials have reported that maintaining normoglycemia with intensive insulin regimen reduces the cardiovascular complications in type 1 diabetes [30]. In this study, the secondary diabetic macrovascular complications such as non-fatal myocardial infarction, stroke/transient ischemic attack, and limb amputations were clustered together to form a composite endpoint and the incidence was similar in both the groups at 3-month post-transplant. There were no secondary events at the rest of the time points. This could be due to a stringent recipient selection or could be due to missing data. In a recent world consensus conference, experts could not draw a conclusion with regards to the effects of PTA on cardiovascular disease progression [31]. The registry does not collect data on modifiable risk factors for coronary artery disease/stroke such as tobacco use, physical activity, blood pressure, and dyslipidaemia. Some of the patients might not have adequate risk factor control. While comparing the incidence of peripheral vascular disease, only amputations were included. The other parameter indicating the progression of vascular disease such as the need for intervention (endovascular or open bypass) was not part of the standard dataset and so not included in our analysis. Hence, in the light of the above, no robust conclusions could be made.

The registry does not record data on diabetic microvascular complications such as retinopathy and neuropathy post-transplantation. Regarding nephropathy, we have compared creatinine post-transplantation between the DBD and DCD recipients and found no significant difference at all time points. There are concerns regarding the risk of accelerated decline in kidney function after a PTA [32, 33]. Even a moderate impairment of kidney function pre-transplant is associated with an increased risk for progression to end stage renal disease after a PTA [34]. Recipients who develop end stage renal failure after a PTA have a three-fold increased risk of mortality [35]. The use of calcineurin inhibitors may contribute to the decline in kidney function. However, improvement in glycaemic control post PTA could reverse the effects of diabetic nephropathy in the longer term [36]. In our study, correlation of creatinine along with proteinuria, concurrent use of ACE (Angiotensin converting enzyme) inhibitors and estimated glomerular filtration rate (eGFR) would have given a better overview about the native renal function. As the rest of the parameters were not part of the standard dataset, we could not correlate them. Future studies could include these parameters and focus on whether DCD grafts have a detrimental effect on the native kidney function after a PTA.

Evaluation of potential differences in HbA1c, weight gain and serum creatinine were performed at each time point separately due to its simple interpretation and its ability to use all the available data. However, per-time point analysis does not consider the overall difference, and inflates the type-1 error rate due to multiple testing. To counteract these deficiencies and the missing data, imputation techniques were necessary and refraining from their use might have led to biased results [37, 38]. Hence, we performed mixed-effects analysis of repeated measures to obtain unbiased results. There was no overall difference in HbA1c, weight gain and serum creatinine between DBD and DCD recipients in the mixed-effects analysis, which further strengthens our study results.

Among the studies reporting survival outcomes of PTA from DCD donors, this study has the highest number of PTA’s performed from DCD donors. This study reports similar 1-, 5-, and 10-year graft and patient survival (unadjusted) for DBD and DCD recipients. In comparison with the previous UK transplant registry analysis by Muthusamy et al. [8], the 1-year graft survival in this study was slightly higher in both the groups, whereas the 1-year patient survival was similar. The slightly higher 1-year graft survival was probably due to a greater number of transplants over time in both the groups. The previously reported higher thrombotic graft loss (statistically insignificant) in DCD group was not observed in this study. Despite significantly improved outcomes and the ability to achieve long term normoglycemia without the risk of hypoglycaemia, PTA is still not widely recognized by healthcare professionals involved in diabetes care [28, 29]. There has been a conservative approach in offering PTA and even more so when it comes to acceptance of DCD grafts. This bias leading to selection of better-quality donors in both the groups could explain the similar survival outcomes observed. Pancreas Donor Risk Index (PDRI) could have been calculated (without using DBD/DCD) to compare the difference between the donors on the other variables but it was not performed as both the groups were comparable except for BMI and also recent literature evidence has questioned the inclusion of race as an indicator of pancreas donor quality [39, 40]. Future studies could shed more light on the outcomes of PTA from extended criteria DCD donors.

There was an observed male-to-female recipient ratio of 1:2 in both DBD and DCD groups. This ratio stands at odds with the proportion of male-to-female incidence of type 1 diabetes mellitus based on the results from large population cohort studies [41, 42]. One plausible explanation for this difference is that the number of female registrations were nearly equal to male registrations in the national pancreas transplant waiting list [43]. The national pancreas allocation policy does not provide any weightage for female patients. We are unable to comment whether this difference might have influenced our results.

Despite the strengths of the study, we acknowledge the following shortcomings. Firstly, this study suffers a bias due to the retrospective nature, that is inherent to all registry analysis; secondly, the sample size is small to perform a multivariate analysis but this would be an issue in most other studies due to the smaller proportion of the patients undergoing PTA especially from DCD donors. Future studies with multinational collaborative data may be able to generate sufficient numbers to allow a robust comparison. Finally, when comparing the metabolic outcomes, hypoglycaemic episodes, concurrent usage of oral hypoglycaemic agents, and other metabolic parameters such as C-peptide and glucose tolerance test were not compared as they were not part of the registry data. Incorporation of the above parameters along with the pancreas extraction times (cross clamp to organ out of the body) in addition to the standardized reporting would be helpful. Future studies could focus on reporting the functional outcomes utilizing the Igls criteria [44].

This is the first study reporting the outcomes of national data on PTA from DCD donors, and also the first study to compare metabolic outcomes alongside survival outcomes between DBD and DCD donors. PTA from DCD donors leads to similar metabolic and long-term survival outcomes to that of transplants from DBD donors. The findings of this study would alleviate the concerns surrounding the use of DCD graft for PTA and thereby supports their usage to expand the donor pool.

## Data Availability

The data analyzed in this study is subject to the following licenses/restrictions: Research data is owned by NHS Blood and Transplant and cannot be provided by the authors. Data can be obtained upon request to NHS Blood and Transplant. Requests to access these datasets should be directed to statistical.enquiries@nhsbt.nhs.uk.

## Abbreviations

ACE, angiotensin converting enzyme; BMI, body mass index; cRF, Calculated reaction frequency; DCD, donation after circulatory death; DBD, donation after brainstem death; eGFR, estimated glomerular filtration rate; HbA_1_c, hemoglobin A_1_c; HLA, human leukocyte antigen; IQR, interquartile range; NHSBT, national health service blood and transplant; NORS, national organ retrieval service; NRP, normothermic regional perfusion; PTA; pancreas transplant alone; UK, United Kingdom; USA, United States of America; WLST, withdrawal of life sustaining treatment.
